# Understanding the impact of acne vulgaris and associated psychological distress on self-esteem and quality of life via regression modeling with CADI, DLQI, and WHOQoL

**DOI:** 10.1038/s41598-023-48182-6

**Published:** 2023-11-30

**Authors:** A. S. M. Morshed, Towhida Noor, Md Ashraf Uddin Ahmed, Fahmida Sultana Mili, Shuma Ikram, Mashiqur Rahman, Shamim Ahmed, Mohammad Borhan Uddin

**Affiliations:** 1 Department of Psychiatry, Dr. Sirajul Islam Medical College, Dhaka, Bangladesh; 2 Department of Psychiatry, Bangladesh Psychiatric Care Limited, Dhanmondi, Dhaka, Bangladesh; 3 Department of Dermatology and Venereology, Bangabandhu Sheikh Mujib Medical University, Dhaka, Bangladesh; 4 Department of Dermatology and Venereology, Matador Diagnostic and Wellness Center, Dhaka, Bangladesh; 5 Department of Medicine, BIRDEM General Hospital, Shahbag, Dhaka, Bangladesh; 6 Department of Obstetrics and Gynecology, Munshiganj General Hospital, Munshiganj, Dhaka, Bangladesh; 7 Department of Pediatrics, East West Medical College and Hospital, Dhaka, Bangladesh; 8 Department of Pharmaceutical Sciences, North South University, Bashundhara, Dhaka, 1229 Bangladesh

**Keywords:** Psychology, Health care

## Abstract

Acne vulgaris (AV) is a psychosomatic disorder and can negatively affect individuals, especially in terms of psychological well-being, self-esteem, and quality of life (QoL). The current study aimed to investigate the association between AV and psychological health, as well as the influence of acne and psychological distress in predicting patients' self-esteem and QoL. This cross-sectional study included 150 patients clinically diagnosed with AV. The severity of acne was measured using GAGS, and following that, patients were instructed to complete the following forms: DASS-21, RSES, CADI, DLQI, and WHOQoL. Female AV patients had significantly higher depression (*p = *0.003, t = 3.025) and anxiety (*p < *0.001, t = 3.683). Pearson's correlation analysis indicated a strong, positive, and significant correlation between having acne and experiencing depression (r = 0.630), anxiety (r = 0.661), and stress (r = 0.758) (*p < *0.001). Multiple regression analysis suggested acne and associated psychological distress had a significant and negative impact on the patient's self-esteem and quality of life. This study highlights the multifaceted consequences of AV and the need to manage its psychological distress. It emphasizes the need for holistic patient care that addresses acne's physical and emotional aspects, with the ultimate goal of enhancing well-being and QoL.

## Introduction

Skin is the body's major interface with the outside world and is regarded as the body's primary public relations tool. Therefore, disorders associated with skin have a negative impact on individuals, especially in acceptance of their own image, mental health, and quality of life (QoL)^1,2^.

Acne vulgaris (AV) is a chronic inflammatory disorder of the pilosebaceous units that causes comedones, inflammatory follicular papules and pustules, and nodular cystic lesions in the sebaceous skin areas of the face and trunk^3^. Approximately 9.4% of the world's population is estimated to be impacted by acne, ranking it as the eighth most common disease on a global scale^4^. The global prevalence of acne in adolescents is reported to range from 28.9 to 91.3%^5^. The prevalence of acne vulgaris among adolescents in Asian countries, specifically China, Malaysia, and Saudi Arabia, was documented to be 33%, 34%, and 56%, respectively^6–8^. Within the context of African nations, Nigeria and Egypt have a prevalence of acne vulgaris among female adolescents above 60%^9,10^. Daily activities have a more significant impact on acne throughout adolescence since this is a time of rapid physical, intellectual, and emotional transformation; therefore, at this age, it can seriously influence their psychological health^11^.

The association between acne and stress has been explained by several psychoneuroendocrinological systems. Both endogenous and external stresses can cause acne or make it worse by activating the cutaneous (local) HPA-like axis and the systemic HPA axis^12^. Previous research has established AV as a substantial risk factor for psychological morbidity in young and older adults. Patients having moderate to severe acne may experience psychological issues due to the negative visual aspects of AV^13^. Also, acne is a psychosomatic disorder, meaning it affects both the body and the psyche^14^. Psychological distress associated with AV includes low self-esteem, social withdrawal, stress, anxiety, depression, frustration, body shame, and family and relationship problems^15,16^. Female patients are psychologically more affected due to AV than male patients^17,18^. In acne sufferers, suicidal ideation was reported to be approximately 6–7%^19,20^, although the exact relationship between acne and suicidal thoughts is still unclear. However, some studies have found that acne can be associated with significant depression and suicidal ideation. For example, a study found that the prevalence of active suicidal ideation among patients with psoriasis and acne was higher than the prevalence reported among general medical patients^19,21^. Another study found that adolescents with acne had increased rates of suicidal ideation and mental health problems^22^.

In addition to the psychological impact, acne can significantly affect an individual's self-esteem and self-image. Self-esteem is defined as "the reasonable or justifiable sense of one's worth or importance"^23^. The development of self-esteem and individuality is critical in adolescents. A noticeable and potentially debilitating skin condition can cause interpersonal rejection and problems with social, professional, and sexual competence, which can have a detrimental effect on psychosocial development and sexual maturity and, ultimately, on QoL^24^. Several national recommendations support the incorporation of health-related quality of life (HRQoL) assessments in acne patients^25^, with the European Dermatology Forum S3-Guideline for the Treatment of Acne proposing adopting a QoL measure as a crucial component of acne care^26^.

The purpose of the current study was to contribute to the literature on dermatology and psychology and investigate the relationship between having severe acne and experiencing psychological distress and how they affect self-esteem and quality of life in affected patients using a regression model-based approach.

## Results

### Sociodemographic characteristics of AV patients

A total of 150 AV patients with a mean age of 21.70 ± 3.09 years were enrolled in the study, and among them, 60% (n = 90) were female. The mean BMI of the patients was 22.56 ± 3.18. The majority (64.7%; n = 97) of the AV patients included in this study were students, and 84.7% (n = 127) had a monthly income of 10,000 or more. Over half of the respondents said they did not have any family history of acne (56.0%; n = 84), 80% (n = 120) had facial acne, with or without truncal involvement, and the majority of the respondents had an onset of acne between 11 and 20 years (62.0%; n = 93). In terms of treatment, most patients were prescribed topical medicines either as single-drug therapy or in combination with systemic medications (94.0%; n = 141). The sociodemographic data of the subjects are presented in Table 1.

**Table 1:** Sociodemographic characteristics and history of participants with acne vulgaris.

	n	%
Gender		
Male	60	40
Female	90	60
Age (mean age: 21.70 ± 3.09 years)		
16 to 20 years	59	39.3
21 to 25 years	58	38.7
26 to 30 years	33	22
BMI (mean score: 22.56 ± 3.18)		
16 to 20	39	26
21 to 25	78	52
26 to 30	33	22
Employment status		
Student	97	64.7
Service holder	29	19.3
Housewife	24	16
Socioeconomic statu**s** (BDT)		
10,000 and less	23	15.3
10,000 to 30,000	67	44.7
30,000 and above	60	40
Marital status		
Unmarried	97	64.2
Married	54	35.8
Habitat		
Urban	116	77.3
Rural	34	22.7
Sleep hours (mean hour: 6.72 ± 1.10 h)		
4 to 6 h	59	39.3
7 to 8 h	84	56
9 to 10 h	7	4.7
Acne affected area		
Face	64	42.7
Trunk	30	20
Face and trunk	56	37.3
Age of acne onset (mean age: 15.91 ± 3.93)		
Before ten years	21	14
Between 11 and 20 years	93	62
After 20 years	36	24
Family history of acne		
Present	66	44
Absent	84	56
Current acne treatment		
None	9	6
Topical	46	30.7
Topical + Systemic	73	48.6
Topical + Systemic retinoid	22	14.7

### Comparison of severity of acne, depression, anxiety, and stress in terms of sociodemographic characteristics of AV patients

The mean GAGS score of the total sample was 25.11 ± 0.61, which was significantly higher in female patients (27.34 ± 0.65) than in male AV patients (21.77 ± 1.05) (t = 4.762, *p < *0.001). In line with this, female AV patients reported significantly higher depression (t = 3.025, *p = *0.003), anxiety (t = 2.055, *p = *0.042), and stress (t = 2.736, *p = *0.007) levels compared to males. AV patients with high BMI had significantly higher anxiety (F(2,147) = 4.371, *p = *0.014) and stress (F(2,147) = 4.277, *p = *0.016). Patients who had a family history of acne had significantly higher GAGS scores (t = 2.527, *p = *0.013), and patients with facial acne had higher levels of depression (F(2,147) = 3.514, *p = *0.032), anxiety (F(2,147) = 8.857, *p < *0.001), and stress (F(2,147) = 3.702, *p = *0.027). The summary of these results is shown in Table 2.

**Table 2:** Comparison of acne severity and depression, anxiety, and stress scores among participants based on sociodemographic variables and family history.

	Global Acne Grading Severity (GAGS)	Depression Anxiety Stress Scale (DASS)		
	Acne severity (mean score ± SEM)	Depression (mean score ± SEM)	Anxiety (mean score ± SEM)	Stress (mean score ± SEM)
Gender				
Male	21.77 ± 1.05	8.90 ± 0.35	7.20 ± 0.37	10.50 ± 0.37
Female	27.34 ± 0.65	10.18 ± 0.25	8.29 ± 0.36	11.84 ± 0.32
p	0.000**	0.003**	0.042*	0.007**
t	4.762	3.025	2.055	2.736
Age				
16 to 20 years	25.81 ± 0.80	9.75 ± 0.31	7.95 ± 0.40	11.36 ± 0.37
21 to 25 years	23.95 ± 1.08	9.21 ± 0.34	7.47 ± 0.43	11.14 ± 0.39
26 to 30 years	25.91 ± 1.47	10.33 ± 0.50	8.36 ± 0.58	11.52 ± 0.60
p	0.323	0.133	0.424	0.839
F	1.138	2.043	0.863	0.176
BMI				
16 to 20	23.41 ± 1.09	9.18 ± 0.37	6.72 ± 0.53	10.28 ± 0.47
21 to 25	25.24 ± 0.79	9.64 ± 0.29	7.99 ± 0.34	11.40 ± 0.31
26 to 30	26.82 ± 1.59	10.30 ± 0.52	8.88 ± 0.57	12.30 ± 0.58
p	0.156	0.188	0.014*	0.016*
F	1.881	1.689	4.371	4.277
Employment status				
Student	24.82 ± 0.70	9.60 ± 0.23	7.79 ± 0.29	11.22 ± 0.28
Service holder	27.52 ± 1.39	10.41 ± 0.51	8.41 ± 0.59	12.21 ± 0.57
Housewife	23.38 ± 1.90	9.04 ± 0.70	7.42 ± 0.90	10.58 ± 0.78
p	0.111	0.147	0.51	0.131
F	2.229	1.945	0.676	2.059
Socioeconomic status				
10,000 and less	25.39 ± 1.38	9.26 ± 0.50	7.96 ± 0.68	11.04 ± 0.58
10,000 to 30,000	24.67 ± 0.96	9.76 ± 0.33	7.90 ± 0.43	11.40 ± 0.39
30,000 and above	25.50 ± 0.98	9.67 ± 0.21	7.77 ± 0.37	11.30 ± 0.37
p	0.812	0.718	0.962	0.886
F	0.208	0.332	0.039	0.121
Habitat				
Urban	25.29 ± 0.72	9.61 ± 0.25	7.93 ± 0.31	11.33 ± 0.29
Rural	24.50 ± 1.17	9.85 ± 0.42	7.59 ± 0.46	11.24 ± 0.44
p	0.59	0.637	0.586	0.876
t	0.539	0.473	0.546	0.157
Sleep hours				
4 to 6 h	26.02 ± 1.17	10.00 ± 0.38	8.20 ± 0.47	11.76 ± 0.45
7 to 8 h	24.64 ± 0.70	9.52 ± 0.26	7.69 ± 0.32	11.11 ± 0.28
9 to 10 h	23.14 ± 2.55	8.57 ± 0.81	6.86 ± 0.99	9.86 ± 1.20
p	0.439	0.294	0.455	0.189
F	0.828	1.234	0.792	1.686
Acne affected area				
Face	26.31 ± 0.97	10.28 ± 0.37	8.92 ± 0.41	12.03 ± 0.38
Trunk	23.93 ± 1.26	9.05 ± 0.31	6.57 ± 0.39	10.40 ± 0.54
Face and trunk	24.38 ± 1.00	9.50 ± 0.39	7.97 ± 0.51	10.96 ± 0.38
p	0.235	0.032*	0.000**	0.027*
F	1.461	3.514	8.857	3.702
Age of acne onset				
Before ten years	25.71 ± 1.95	9.95 ± 0.50	9.33 ± 0.63	12.19 ± 0.75
11 to 20 years	24.85 ± 0.77	9.62 ± 0.27	7.26 ± 0.34	11.20 ± 0.30
After 20 years	25.44 ± 1.17	9.61 ± 0.45	8.53 ± 0.48	11.06 ± 0.51
p	0.855	0.865	0.009**	0.341
F	0.157	0.146	4.854	1.084
Family history of acne				
Present	26.83 ± 1.00	9.65 ± 0.30	8.03 ± 0.41	11.39 ± 0.36
Absent	23.76 ± 0.74	9.68 ± 0.30	7.71 ± 0.34	11.24 ± 0.34
p	0.013*	0.95	0.552	0.754
t	2.527	0.063	0.597	0.314

* *p < *0.05; ** *p < *0.01.

t = Independent samples *t*-test; F = One-way ANOVA, Bonferroni post hoc analysis.

Bold values indicate significant differences.

### Correlation analysis between acne and depression, anxiety and stress

An inspection of histograms suggested that the assumption of normality was not violated (Supplementary Fig. 1). Similarly, Shapiro–Wilk tests suggested that acne severity, *W*(150) = 0.990, *p = *0.329, depression, *W*(150) = 0.983, *p = *0.056, anxiety, *W*(150) = 0.986, *p = *0.130, and stress, *W* (150) = 0.985, *p = *0.102, were normally distributed. Additionally, an inspection of scatterplots suggested a linear relationship between acne severity and depression, anxiety, and stress and that the assumption of homoscedasticity was not violated (Supplementary Fig. 2). A Pearson's correlation analysis indicated a strong, positive, and significant correlation between acne and depression (r = 0.630, *p < *0.001), anxiety (r = 0.661, *p < *0.001), and stress (r = 0.758, *p < *0.001). These data are presented in Table 3.

**Table 3:** Correlation matrix for acne vulgaris and presence of psychiatric comorbidities.

	Acne	Depression	Anxiety	Stress
Acne	–	0.630**	0.661**	0.758**
Depression	0.630**	–	0.669**	0.656**
Anxiety	0.661**	0.669**	–	0.712**
Stress	0.758**	0.656**	0.712**	–

**Correlation is significant at the 0.01 level (2-tailed); N = 150.

GAGS, Global acne grading system; DASS, Depression anxiety stress scale, *W*, Shapiro–Wilk statistics.

### Multiple linear regression analysis for self-esteem levels in AV patients

A multiple linear regression analysis was conducted to examine whether self-esteem level can be predicted by the severity of acne, depression, anxiety, and stress. The model was significant, F(8,141) = 21.555, *p < *0.001, explaining 55.0% (R^2^ = 0.550) of the variance in the outcome variable. In addition, coefficients were further assessed to ascertain the influence of each of the factors on the criterion variable (self-esteem). The results showed that acne (B = − 0.396, t = − 4.760, *p < *0.001) and anxiety (B = − 0.385, t = − 2.007, *p = *0.047) had a significant and negative impact on self-esteem. The results are summarised in Table 4.

**Table 4:** Multiple linear regression analysis showing the effect of acne and associated psychiatric morbidities on the prediction of self-esteem^ψ^.

	Unstandardised Coefficients		β	t	p	95% CI		Collinearity Statistics	
	B	SE				Lower Bound	Upper Bound	Tolerance	VIF
Gender^a^	-1.125	0.851	− 0.082	− 1.322	0.188	− 2.807	0.557	0.833	1.200
Age	− 0.058	0.124	− 0.026	− 0.463	0.644	− 0.303	0.188	0.981	1.020
Family history of acne^b^	− 0.562	0.812	− 0.041	− 0.692	0.490	− 2.167	1.042	0.892	1.121
Sleep hours	0.198	0.385	0.030	0.513	0.609	− 0.564	0.959	0.929	1.076
GAGS									
Acne	− 0.396	0.083	− 0.440	-4.760	< 0.001	− 0.560	− 0.231	0.373	2.683
DASS									
Depression	0.071	0.217	0.027	0.326	0.745	− 0.358	0.500	0.457	2.186
Anxiety	− 0.385	0.192	− 0.183	− 2.007	0.047	− 0.763	− 0.006	0.384	2.603
Stress	− 0.388	0.221	− 0.173	-1.758	0.081	− 0.825	0.048	0.329	3.040

GAGS, Global acne grading system; DASS, Depression anxiety stress scale; RSES, Rosenberg self-esteem scale; CI, Confidence interval; B, Unstandardised Coefficients; SE, Standard error of the estimate; β, Standardised Coefficients; VIF, Variance inflation factor.

^a^1: Male; 2: Female.

^b^0: No; 1: Yes.

^ψ^ N = 150, R^2^ = 0.550, F(8,141) = 21.555, *p < *0.001.

### Factors affecting quality of life in AV patients

The mean CADI, DLQI, and WHOQoL score of the total sample was 4.57 ± 0.15, 8.20 ± 0.27, and 71.92 ± 1.39, respectively, and measures from all three scales suggested that QoL was significantly more impaired in female patients (CADI: t = 6.173, *p < *0.001; DLQI: t = 4.438, *p < *0.001; WHOQoL: t = 2.526, *p = *0.013). Location of acne significantly impacted QoL, especially when AV patients had acne on their faces (CADI: F(2,147) = 10.164, *p < *0.001; WHOQoL: F(2,147) = 5.350, *p = *0.006). In addition, QoL was significantly affected in patients with a family history of acne (CADI: t = 2.643, *p = *0.009; DLQI: t = 2.183, *p = *0.031; WHOQoL: t = 2.397, *p = *0.018). AV patients with higher BMI had poorer QoL (F(2,147) = 4.844, *p = *0.009) according to the WHOQoL scale. Results are summarised in Table 5.

**Table 5:** Comparison of quality of life of acne patients with the help of three instruments- Cardiff acne disability index (CADI), Dermatology life quality index (DLQI), and World Health Organization quality-of-life (WHOQoL).

	Quality of life		
	CADI (mean score ± SEM)	DLQI (mean score ± SEM)	WHOQoL (mean score ± SEM)
**Gender**			
Male	3.58 ± 0.24	6.83 ± 0.44	76.15 ± 2.18
Female	5.23 ± 0.15	9.11 ± 0.30	69.10 ± 1.76
p	0.000**	0.000**	0.013*
t	6.173	4.438	2.526
Age			
16 to 20 years	4.61 ± 0.18	8.03 ± 0.37	71.03 ± 2.00
21 to 25 years	4.38 ± 0.25	8.05 ± 0.43	74.08 ± 2.32
26 to 30 years	4,85 ± 0.38	8.76 ± 0.69	69.70 ± 3.29
p	0.48	0.543	0.44
F	0.738	0.614	0.824
BMI			
16 to 20	4.10 ± 0.24	7.18 ± 0.44	77.49 ± 2.33
21 to 25	4.68 ± 0.18	8.47 ± 0.35	71.96 ± 1.89
26 to 30	4.88 ± 0.41	8.76 ± 0.69	65.25 ± 3.27
p	0.141	0.069	0.009**
F	1.987	2.72	4.844
Sleep hours			
4 to 6 h	4.66 ± 0.28	8.31 ± 0.49	71.29 ± 2.43
7 to 8 h	4.54 ± 0.17	8.26 ± 0.32	72.10 ± 1.77
9 to 10 h	4.29 ± 0.68	6.57 ± 0.84	75.00 ± 5.46
p	0.838	0.403	0.855
F	0.177	0.914	0.157
Acne affected area			
Face	5.17 ± 0.24	8.88 ± 0.44	67.83 ± 2.48
Trunk	3.50 ± 0.30	7.30 ± 0.65	79.79 ± 2.47
Face and trunk	4.46 ± 0.18	7.91 ± 0.35	72.38 ± 1.83
p	0.000**	0.065	0.006**
F	10.164	2.79	5.35
Age of acne onset			
Before ten years	4.86 ± 0.51	8.38 ± 0.90	70.24 ± 3.93
11 to 20 years	4.55 ± 0.18	8.06 ± 0.33	72.42 ± 1.81
After 20 years	4.47 ± 0.27	8.44 ± 0.51	71.61 ± 2.63
p	0.722	0.81	0.865
F	0.327	0.211	0.146
Family history			
Present	5.02 ± 0.25	8.86 ± 0.44	68.09 ± 2.38
Absent	4.23 ± 0.17	7.68 ± 0.32	74.93 ± 1.58
p	0.009**	0.031*	0.018*
t	2.643	2.183	2.397

*p < 0.05; **p < 0.01.

t = Independent samples *t*-test; F = One-way ANOVA, Bonferroni post hoc analysis.

Bold values indicate significant differences.

### Multiple linear regression analysis for QoL in AV patients

The dependent variable (QoL) was regressed in predicting variables of acne, depression, anxiety, stress, and self-esteem. The independent variables significantly predicted the quality of life (CADI: R^2^ = 0.742, F(9,140) = 44.819, DLQI: R^2^ = 0.704, F(9,140) = 37.018, WHOQoL: R^2^ = 0.632, F(9,140) = 26.682; *p < *0.001), which indicates that the five factors under study had a significant impact on quality of life.

Additionally, coefficients were further assessed to ascertain the influence of each of the factors on the criterion variable (QoL). According to the CADI and DLQI, having more severe acne (CADI: B = − 0.159, t = -8.841, *p < *0.001; DLQI: B = − 0.213, t = -6.039, *p < *0.001), high depression (DLQI: B = − 0.180, t = − 2.109, *p = *0.037) and stress (DLQI: B = − 0.207, t = − 2.353, *p = *0.020), and low self-esteem (CADI: B = 0.054, t = 3.158, *p = *0.002) predicted more impairment in QoL. Results from WHOQoL scale showed that acne (B = − 0.341, t = -1.663, *p = *0.048), depression (B = -1.723, t = -3.466, *p = *0.001), and anxiety (B = -1.226, t = − 2.752, *p = *0.007) had a significant and negative impact on QoL, while self-esteem had a significantly positive impact on QoL (B = 0.520, t = 2.693, *p = *0.008). The results are presented in Table 6.

**Table 6:** Multiple linear regression analysis showing the effect of acne, associated psychiatric morbidities, and self-esteem on the prediction of quality of life (QoL) using three different scales- Cardiff acne disability index (CADI), Dermatology life quality index (DLQI), and World Health Organization quality-of-life (WHOQoL).

		Unstandardised Coefficients		β	t	p	95% CI		Collinearity Statistics	
		B	SE				Lower Bound	Upper Bound	Tolerance	VIF
CADI^†^	Gender^a^	− 0.215	0.172	− 0.059	− 1.245	0.215	− 0.556	0.126	0.823	1.215
	Age	0.021	0.025	0.036	0.822	0.413	− 0.029	0.070	0.979	1.021
	Family history of acne^b^	0.170	0.164	0.047	1.041	0.300	− 0.153	0.494	0.889	1.125
	Sleep hours	0.058	0.078	0.034	0.753	0.453	− 0.095	0.212	0.928	1.078
	*GAGS*									
	Acne	− 0.159	0.018	− 0.669	-8.841	< 0.001	− 0.195	− 0.124	0.321	3.114
	DASS									
	Depression	− 0.002	0.044	− 0.003	− 0.050	0.960	− 0.089	0.084	0.457	2.188
	Anxiety	− 0.020	0.039	− 0.035	− 0.501	0.617	− 0.097	0.058	0.374	2.677
	Stress	0.049	0.045	0.082	1.089	0.278	− 0.040	0.138	0.322	3.107
	RSES									
	Self-esteem	0.054	0.017	0.202	3.158	0.002	0.020	0.087	0.450	2.223
DLQI^ф^	Gender^a^	− 0.377	0.337	− 0.057	− 1.120	0.265	− 1.043	0.289	-	-
	Age	0.078	0.049	0.074	1.601	0.112	− 0.018	0.175	-	-
	Family history of acne^b^	0.055	0.320	0.008	0.172	0.864	− 0.577	0.687	-	-
	Sleep hours	0.100	0.152	0.032	0.662	0.509	− 0.200	0.400	-	-
	GAGS									
	Acne	− 0.213	0.035	− 0.490	− 6.039	< 0.001	− 0.282	− 0.143	–	–
	DASS									
	Depression	− 0.180	0.085	− 0.143	− 2.109	0.037	− 0.349	0.011	–	–
	Anxiety	0.016	0.076	0.016	0.207	0.997	− 0.135	0.167	–	–
	Stress	− 0.207	0.088	− 0.191	− 2.353	0.020	− 0.381	0.033	–	–
	RSES									
	Self-esteem	0.055	0.033	0.114	1.660	0.099	0.011	0.120	–	–
**WHOQoL**^**ψ**^	Gender^a^	4.927	1.962	0.142	2.511	0.013	1.048	8.807	–	–
	Age	0.009	0.285	0.002	0.032	0.974	− 0.554	0.572	–	–
	Family history of acne^b^	-1.489	1.864	− 0.043	− 0.799	0.426	− 5.173	2.195	–	–
	Sleep hours	− 0.763	0.884	− 0.046	− 0.863	0.390	− 2.510	0.985	–	–
	GAGS									
	Acne	− 0.341	0.205	− 0.151	− 1.663	0.048	− 0.747	0.064	–	–
	DASS									
	Depression	-1.723	0.497	− 0.263	− 3.466	0.001	− 2.707	− 0.740	–	–
	Anxiety	-1.226	0.445	− 0.231	− 2.752	0.007	− 2.106	− 0.345	–	–
	Stress	− 0.635	0.512	− 0.112	− 1.241	0.217	− 1.648	0.377	–	–
	RSES									
	Self-esteem	0.520	0.193	0.206	2.693	0.008	0.138	0.901	–	–

Abbreviations: GAGS, Global acne grading system; DASS, Depression anxiety stress scale; RSES, Rosenberg self-esteem scale; CADI, Cardiff Acne Disability Index; DLQI, Dermatology Life Quality Index; WHOQoL, World Health Organization quality of life; CI, Confidence interval; B, Unstandardised Coefficients; SE, Standard error of the estimate; β, Standardised Coefficients; VIF, Variance inflation factor.

^a^1: Male; 2: Female.

^b^0: No; 1: Yes.

^**†**^ N = 150, R^2^ = 0.742, F(9,140) = 44.819, *p < *0.001.

^**ф**^ N = 150, R^2^ = 0.704, F(9,140) = 37.018, *p < *0.001.

^**ψ**^ N = 150, R^2^ = 0.632, F(9,140) = 26.682, *p < *0.001.

## Discussion

To the best of our knowledge, the present study is the first comprehensive study conducted in Bangladesh that addresses the strength and relationship between acne and depression, anxiety and stress, as well as the impact of acne and associated mental illness on patients' self-esteem and QoL. While there have been previous studies conducted globally that have yielded similar results, this particular study focuses on a specific population in Bangladesh. By adopting a localised approach, this research aims to uncover valuable insights into the distinct regional factors that may impact the associations between psychological scales and acne. Local variations in diet, healthcare systems, and environmental conditions can play a significant role in how acne is experienced and managed, as well as how psychological distress manifests. Our research offers a specific regional perspective, contributing to a nuanced understanding of the impact of acne on individuals in Bangladesh.

In this cross-sectional study, the QoL of AV patients was assessed using three instruments: CADI (acne-specific), DLQI (dermatology-specific), and WHOQoL (psychology-specific). Rosenberg's self-esteem scale (RSES) was utilized to assess the self-esteem in AV patients. We rigorously assessed the internal consistency of the questionnaires used in our study to ensure data reliability. The CADI demonstrated a Cronbach's alpha coefficient of 0.757, aligning with prior studies reporting values ranging from 0.68 to 0.839^27,28^. The DLQI had a Cronbach's alpha of 0.823, consistent with Jorge et al.'s findings (α = 0.90)^29^. The WHOQOL exhibited strong internal consistency with a Cronbach's alpha of 0.801, similar to the work of Williams et al. (psychological domain, α = 0.81)^30^. The RSES, measuring self-esteem, showed a Cronbach's alpha of 0.783, in line with Lima-Castro et al.'s research (α = 0.86)^31^.

The severity of acne in the current study was assessed by a dermatologist with the help of GAGS, while DASS-21 was used to measure the psychological distress of AV patients. Regarding the DASS-21, our study found strong internal consistency across its subscales, with Cronbach's alpha values of 0.791 for depression, 0.787 for anxiety, and 0.831 for stress. These results align with the study conducted by Sagaltici and Tas^32^, which similarly reported robust Cronbach's alpha coefficients for the DASS-21 subscales in assessing mental health.

Although the mean scores for both CADI (4.57 ± 0.15; maximum possible score 15) and DLQI (8.20 ± 0.27; maximum possible score 30) were pretty low, our findings confirm that acne significantly impairs self-esteem and QoL. The main reason for comparatively low scores could be the high prevalence of mild to moderate acne (78.0%, n = 117) in community settings.

The interaction between acne and psychosocial issues is complex and can result in negative emotional responses like depression, anxiety, stress, helplessness and frustration, and even suicidal thinking, which can impact AV patients' quality of life and self-esteem^13,33,34^. Additionally, a number of previous studies reported that AV patients experienced noticeably higher psychological stress^35–39^. According to data from a national survey of young people's health done in New Zealand (Youth 2000), acne was linked to a higher likelihood of depressive symptoms, anxiety, and suicide attempts^40^. Findings from our study suggest that there is a strong, positive, and significant relationship between acne patients and depression, anxiety, and stress, which is in agreement with the majority of the studies that explored the correlation between having acne and experiencing depression^41^, anxiety^41,42^, and stress^43^. Moreover, a recent meta-analysis that reviewed 42 studies reported a possible association between acne and depression and anxiety^44^. However, few studies indicate no significant correlation between acne and feeling anxious or depressed^32,45^. The disparities in these results could be caused by variations in the experimental design and the demography of the sample.

More than half of the patients who participated in our research had acne when they were 11–20 years old (n = 93; 62.0%), and this is in line with a previous finding that 85% of acne cases begin between the ages of 12 and 24^46^. The hormonal surge that occurs before and throughout puberty is most likely to be blamed for this onset.

The mean CADI score of this study is in line with the result from a previous study conducted in Egypt (4.95)^47^, somewhat lower than studies conducted in Iran (7.57), Malaysia (5.1), and India (6.22)^48–50^, but relatively higher than results observed from studies conducted on Montenegrin (3.53), Serbian (3.57 and 2.87), Scottish (1.9), and Nigerian population (3.4)^51–55^. A meta-analysis conducted by Olsen et al. reported the impact of different skin conditions on QoL, where it was stated that the mean overall CDLQI score for acne (5.3) was in sixth place after scabies (9.2), urticaria (7.1), atopic eczema (8.5), psoriasis (8.0), and vitiligo (6.5)^56^.

In the present study, we have found female patients had a more severe form of acne than males, which is consistent with previous research findings^57–59^, and this female predominance may be due to the hormonal changes during menstruation or increased levels of stress among females^47^. In accordance with this, girls were reported to experience a greater impact on QoL, as evident from the statistically significant difference in the mean CADI, DLQI, and WHOQoL scores. Additionally, DASS-21 scores indicated that the psychological impact of AV was higher in females than it was in males. Similar to these findings, several other studies found that girls are more vulnerable to the negative psychological impact of AV than boys^17,51,52,60–64^. However, Tasoula et al.^33^ and Tayel et al.^47^ found no differences in QoL between male and female patients affected with AV.

Among the AV patients included in this study, significantly decreased self-esteem was observed in female patients (26.08 ± 0.68) compared to male patients (29.10 ± 0.87) (t = 2.715, *p = *0.008). Most studies revealed that women with acne were more likely to experience higher levels of self-consciousness and self-perceived stress, lower levels of self-esteem and self-worth, lower levels of body satisfaction, lower levels of self-attitude, and higher levels of feelings of helplessness^23,47^.

The duration and quality of sleep have been found to exert a notable influence on the severity of acne. In a recent study, Schrom et al. reported that poor sleep quality and short sleep duration may be associated with worse acne severity in adults^65^. Another study investigating the relationship between sleep quality and acne severity found that sleeping too late can cause a person to lack sleep, which can cause an increase in inflammatory factors and affect the incidence and exacerbation of acne^66^. However, in our study, we did not notice any significant difference in acne severity among the sub-groups categorized by their sleep duration.

Since acne on the face is frequently noticeable, it can heighten concerns with socialization and body image. Thus, it is not surprising that someone with facial acne may experience severe psychological disability^67^. Our study also confirms that patients with facial acne suffer from a higher level of psychological distress, consequently affecting their QoL. Another remarkable finding of this study is that patients having a family history of acne reported more impairment in QoL. Previously, not many studies analyzed the correlation between family history and QoL in patients with AV. A recent study from China^68^ reported that patients with skin conditions (including acne) and family history were more likely to experience QoL impairment by 2.221 times (95% CI 1.333–3.703) than patients without a family history. In the same study, patients with acne had a 1.219 (95% CI 0.589–2.520) times higher risk of QoL impairment than those with psoriasis. Durai et al.^69^ found a significant relationship between family history of AV and CADI/DLQI scores and came to the conclusion that family history affected QoL, whereas Đurović et al.^51^ did not find any statistically significant association between family history and CADI/DLQI scores.

This study also found that acne, depression, anxiety, and stress were significant negative predictors of self-esteem and QoL. Numerous studies reported that moderate to severe acne correlated to low self-esteem and poor QoL^70–72^. Vilar et al.^73^ highlighted that patients with more severe acne experienced more impairment in QoL; however, the same study did not find any significant association between acne severity and self-esteem. Desai et al.^74^ and El-Hamad et al.^10^ examined the relationship between the severity of acne and QoL in school-aged adolescents and found a position correlation between the variables. Self-esteem, on the other hand, is positively associated with QoL or, in other words, psychological well-being. Individuals with high self-esteem have good mental health, high psychological resilience, and strong personal and social relationships, which allow them to have a better standard of living and high QoL compared to the patients with decreased self-esteem^75^. Additionally, having high self-esteem could serve as a protective factor when coping with chronic illness and challenges^76^.

### Limitations

As a cross-sectional study, it captures data at a single point in time, making it challenging to determine the direction of causality between acne and psychological factors. While the study reveals associations and relationships between these variables, it cannot infer causation. The design's static nature limits our ability to assess how changes over time may influence the observed associations. Future longitudinal research is required to unravel the temporal aspects of these relationships and provide a more robust understanding of causation.

Moreover, the COVID-19 pandemic has had a significant impact on mental health worldwide, and it is possible that it may have affected the results of our study on the psychological impact of AV. The pandemic has led to increased stress, anxiety, and depression, which could exacerbate the psychological distress associated with AV. Additionally, the pandemic has disrupted healthcare services, leading to delays in diagnosis and treatment, which could further impact patients' quality of life and self-esteem. However, further studies are needed to understand the impact of the pandemic on acne patients' mental health and quality of life.

It is also worth mentioning that the psychological status, self-esteem level, and quality of life were measured through self-reporting questionnaires, which may introduce biases associated with self-reporting, such as misclassification and under- or over-reporting information. A systematic diagnosis through interviews of the patients would have provided a more accurate evaluation of the condition.

## Methods

### Study design and selection of participants

This cross-sectional study was conducted between March 2021 and February 2022 in Dhaka, Bangladesh, by the dermatology and psychiatry departments of the BIRDEM General Hospital and Dr. Sirajul Islam Medical College and Hospital. One hundred fifty patients who were diagnosed with AV in the age group of 16 to 30 years were included in the study. The calculation of the sample size was performed using G*Power version 3.2. Based on a statistical power of 80% and an acceptable alpha error rate of 5%, the minimum sample size required for this study was calculated to be 118 participants. Eventually, a total of 150 patients who satisfied the specified inclusion criteria and provided their informed consent were included in this study.

Patients with other dermatological conditions, previous history of cutaneous or aesthetic injury or scarring, cognitive impairment, pregnancy, breastfeeding, patients who were on immunosuppressives, corticosteroids, or medications that might affect cognition, and patients suffering from serious medical conditions (e.g., cardiovascular or pulmonary disorder) were excluded from the research.

### Study tools

#### Sociodemographic and patient history form

The form was prepared by the researchers in order to collect sociodemographic information (gender, age, BMI, employment, socioeconomic status, marital status, and habitat) and clinical data (sleep hours, acne-affected area, age of acne onset, family history of acne, and current acne treatment), to aid in the analysis process.

#### Global acne grading system (GAGS)

The global acne grading system (GAGS) was developed to measure the severity of acne in patients^77^. Briefly, GAGS takes into account six areas of the face and chest/upper back with a factor for each area based on the surface area, distribution, and density of pilosebaceous units (forehead = 2, right cheek = 2, left cheek = 2, nose = 1, chin = 1, chest and upper back = 3). Depending on the type and severity of the lesions, each location was graded (No lesion = 0, One comedone = 1, Papule = 2, One pustule = 3, One nodule = 4). The local score was then calculated by multiplying factors and grade and finally summed to grade acne severity.

#### Depression anxiety stress scale-21 (DASS-21)

The Depression anxiety stress scale-21 (DASS-21) is basically a 21-item self-report questionnaire that is designed to assess the severity of depression, anxiety, and stress symptoms. Each item of the DASS-21 corresponds to one of the three subscales (depression, anxiety, and stress), with seven items per subscale. The 4-point Likert scale measures symptoms from the past week and ranges from 0 (never) to 3 (nearly usually)^78,79^. Cronbach's alpha coefficient indicated that internal consistency was acceptable for depression (α = 0.791), anxiety (α = 0.787), and stress (α = 0.831) subscales, as well as for the scale overall (α = 0.861).

#### Rosenberg's self-esteem scale (RSES)

Rosenberg's self-esteem scale (RSES) is a 10-item instrument that was developed by Rosenberg in 1965 with the aim of assessing overall self-worth by measuring the positive and negative feelings about oneself^80^. The 4-point Likert scale ranges from 1 (strongly agree) to 4 (strongly disagree) for measuring negative self-esteem (Question-3, 5, 8, 9, and 10) and from 4 (strongly agree) to 1 (strongly disagree) for assessing positive self-esteem (Question-1, 2, 4, 6, 7). Overall, RSES values range between 0 and 40. In our study, Cronbach's alpha for RSES was 0.783.

#### Quality of life

**The Cardiff Acne Disability Index (CADI)** is a 5-item acne-specific questionnaire that was designed to measure acne-induced disability. This 4-point Likert scale ranges from 3 to 0, with the highest total possible score of 15, and assesses the following feelings from the past month: the feeling of aggression/ frustration/ embarrassment, social life and personal relationships, bodily appearance, and perceived acne severity. A higher score indicates more QOL impairment^81^. The Cronbach's alpha for CADI in our study was 0.757.

**The Dermatology Life Quality Index (DLQI)** is a brief skin-specific questionnaire consisting of 10 questions that measure patients' QoL: acne symptoms, feelings, relationships, work and study, sleep, leisure activities, and treatment. The responses are scored from 3 to 0, with a maximum possible score of 30^82^. A higher DLQI score means more impairments in QoL. The Cronbach's alpha for DLQI was 0.823.

**The World Health Organization quality-of-life scale (WHOQoL)** is a 26-item self-report questionnaire consisting of four domains (physical health- 7 items, psychological- 6 items, social relationships- 3 items, and environment- 8 items) along with QoL and general health items. The 5-point Likert scale measures symptoms from the past two weeks and ranges from 1 to 5^83,84^. For this study, the psychological domain was used to assess the AV patient's QoL. The six items of this subscale focused on self-image and appearance, positive and negative feelings, ability to concentrate and learn, self-esteem, mentality, and spirituality/religion/personal beliefs^83^. The score obtained for every individual was then transformed to a 0 to 100 scale using the following formula:

$$Transformed\, Scale= \left[\frac{(Actual\, raw \,score-Lowest\, possible\, raw\, score)}{Possible \,raw\, score\, range}\right] \times 100$$

where "actual raw score" is the value that occurred through summation, "lowest possible raw score" is the lowest possible value that could be achieved through summation, and "possible raw score range" is the difference between the highest possible raw score and lowest possible raw score. The lower score means that the more QoL is impaired. The Cronbach's alpha for the psychological domain was 0.801, meaning the subscale used had adequate internal consistency.

### Ethical approval

Ethical approval was acquired from the Institutional Ethics Committee of BIRDEM General Hospital, Dhaka, Bangladesh (project code: BADAS-ERC/EC/19/00281; approval date: 28 December 2019), and the study was carried out in conformity with the ethical principles of the Declaration of Helsinki^85^. During the interview, individuals assigned to collect the responses first explained the purpose and importance of the study to the potential respondents, and their informed consent was obtained.

### Data collection

Dermatologists with AV expertise were responsible for recording demographic information and examining patients' skin. Following that, individuals were sent to an experienced psychiatrist who explained the methodology and scales used in the study. All participants were given a thorough explanation of the study's objectives, as well as specific instructions on how to fill the various scales that would be utilized in the research.

### Statistical analysis

Descriptive statistics were expressed as frequencies and percentages for categorical variables, and for continuous variables, data were presented as mean ± SD, Standard deviation (age, BMI, sleep hours, and mean age of acne onset), or mean ± SEM, Standard Error of Mean. Independent sample *t-*test and one-way ANOVA (Bonferroni correction) were utilized to compare means between groups. Normality tests were performed using the Shapiro–Wilk test and histograms. Pearson's correlation test was used to measure the strength and relationship between acne severity and depression, anxiety, and stress. Multiple linear regression models were used with the enter method to find out potentially predictive factors for self-esteem and QoL. Assumption tests for linearity, homoscedasticity, and multicollinearity were conducted prior to regression analysis (Supplementary Fig. 3–5).

Statistical analysis was performed with IBM Statistical Package for Social Science, SPSS version 25.0 (SPSS Inc., Chicago, IL, USA). p-value less than 0.05 was considered statistically significant.

### Supplementary Information

Supplementary Information.

## Data Availability

The datasets generated during and/or analyzed during the current study are available from the corresponding author upon reasonable request.

## Abbreviations

AV: Acne vulgaris
QoL: Quality of life
WHOQoL: World health organization quality-of-life
CADI: Cardiff acne disability index
DLQI: Dermatology life quality index
RSES: Rosenberg's self-esteem scale
GAGS: Global acne grading system
DASS: Depression anxiety stress scale
