# Supplementary material for: Understanding the impact of acne vulgaris and associated psychological distress on self-esteem and quality of life via regression modeling with CADI, DLQI, and WHOQoL
Source: Sci Rep. 2023 Nov 30;13:21084. doi: 10.1038/s41598-023-48182-6 (PMC10687248; doi:10.1038/s41598-023-48182-6)
Supplement: Supplementary file 1 — Supplementary Information. [file 41598_2023_48182_MOESM1_ESM.pdf]

A regression model- based approach using CADI, DLQI and WHOQoL in acne vulgaris and associated psychological distress impact on patient's self-esteem and quality of life

ASM Morshed[1,6], Towhida Noor[2,7], Md Ashraf Uddin Ahmed[3], Fahmida Sultana Mili[4], Shuma Ikram[5], Mashiqur Rahman[8], Shamim Ahmed[6,8], Mohammad Borhan Uddin[8*]

[1]Department of Psychiatry, Dr. Sirajul Islam Medical College, Dhaka, Bangladesh

[2]Department of Dermatology and Venereology, Bangabandhu Sheikh Mujib Medical University, Dhaka, Bangladesh

[3]Department of Medicine, BIRDEM General Hospital, Shahbag, Dhaka, Bangladesh

[4]Department of Obstetrics and Gynecology, Munshiganj General Hospital, Munshiganj, Dhaka, Bangladesh

[5]Department of Pediatrics, East West Medical College and Hospital, Dhaka, Bangladesh

[6]Department of Psychiatry, Bangladesh Psychiatric Care Limited, Dhanmondi, Dhaka, Bangladesh

[7]Department of Dermatology and Venereology, Matador Diagnostic and Wellness Center, Dhaka, Bangladesh

[8]Department of Pharmaceutical Sciences, North South University, Bashundhara, Dhaka, Bangladesh

Correlation analysis between having acne and experiencing depression, anxiety, and stress

Assumptions

An inspection of histograms suggested that the assumption of normality was not violated (Fig. 1a-d). Similarly, Shapiro-Wilk tests suggested that acne severity, $W(150) = 0.990$, p = 0.329, depression, $W(150) = 0.983$, p = 0.056, anxiety, $W(150) = 0.986$, p = 0.130, and stress, $W(150) = 0.985$, p = 0.102, were normally distributed. Additionally, an inspection of scatterplots suggested a linear relationship between acne severity and depression, anxiety, and stress and that the assumption of homoscedasticity was not violated (Fig. 2a-c).

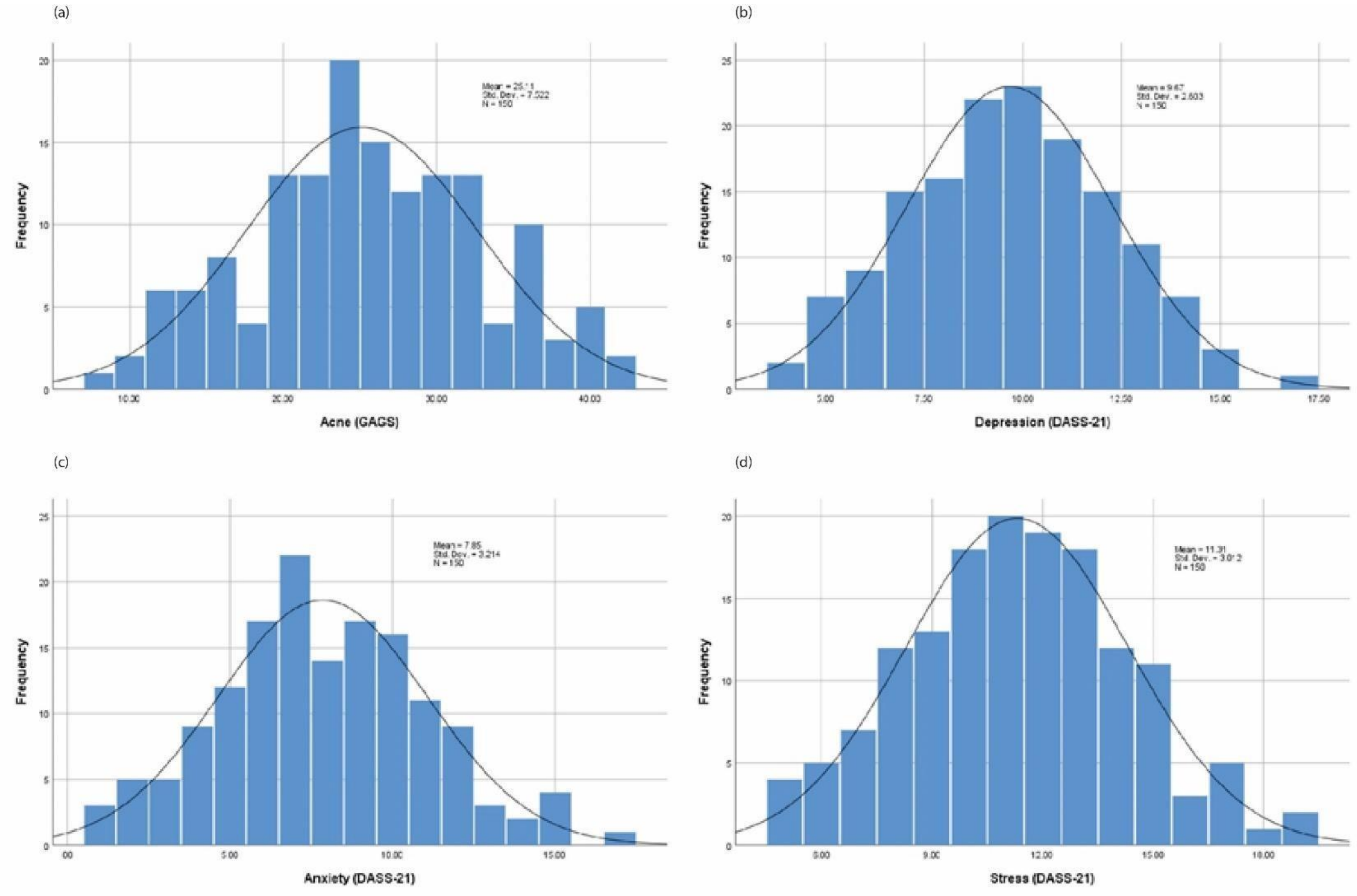

Figure 1. Assumption test for Pearson's correlation analysis between having acne and experiencing depression, anxiety, and stress. Inspection of histograms suggested that the assumption of normality was not violated for acne (a), depression (b), anxiety (c), and stress (d).

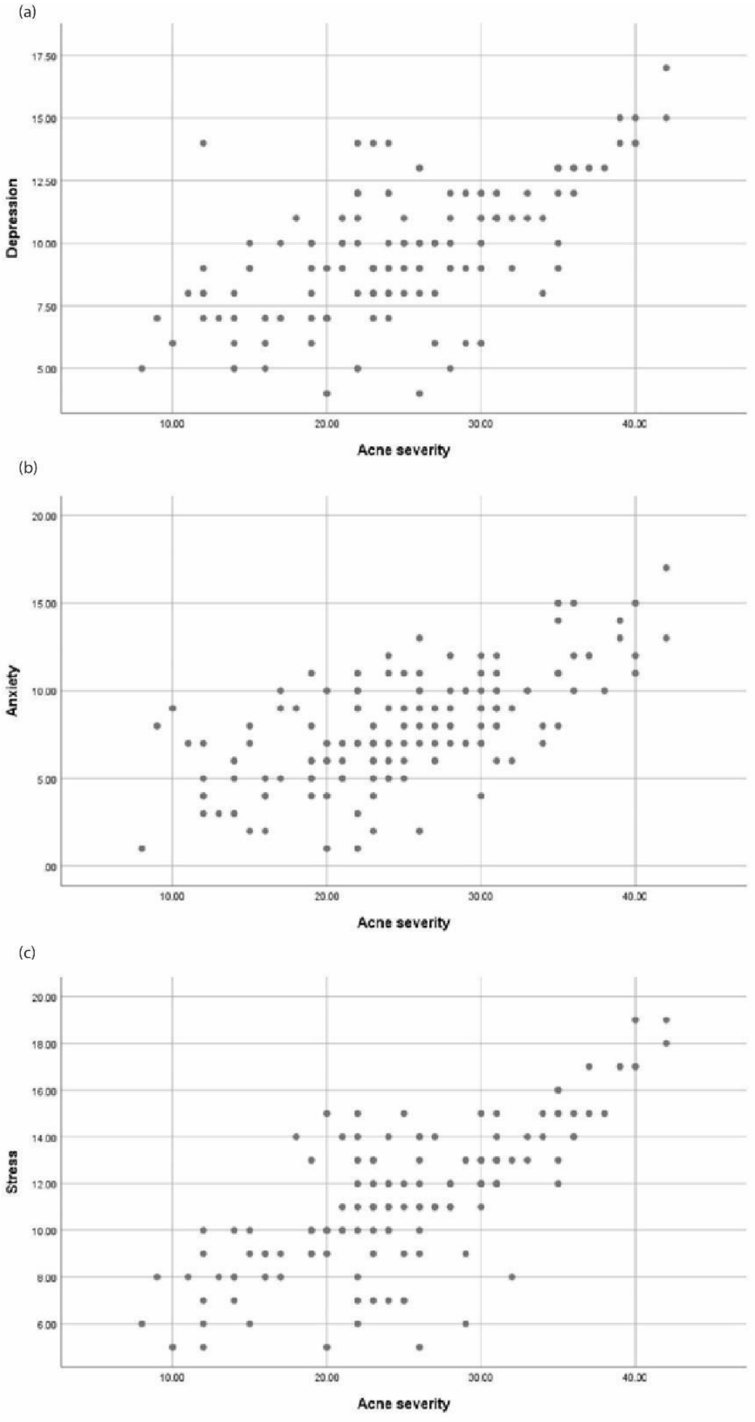

Figure 2. Assumption test for Pearson's correlation analysis between having acne and experiencing depression, anxiety, and stress. Inspection of scatterplots suggested a linear relationship between acne severity and depression (a), anxiety (b), and stress (c) and that the assumption of homoscedasticity was not violated.

Multiple linear regression analysis for self-esteem levels in AV patients

Assumptions

To ensure that there was no multicollinearity, a collinearity diagnostic was performed. The tolerance and variance inflation factor (VIF) values did not indicate a violation of this assumption (Table 4). Durbin-Watson statistic was calculated to assess the assumption that the values of the residuals are independent, which suggested that this assumption was not violated (1.682). A scatterplot was created to assess the assumption that the variance of the residuals was constant (homoscedasticity), and the plot did not indicate a violation of this assumption (Fig. 3a). Moreover, a P-P plot was created to check the assumption that the values of residuals were normally distributed. The plot did not indicate a violation of this assumption (Fig. 3b). Cook's Distance values were calculated to ensure that no influential cases were biasing the model. All values were below 1, suggesting that no cases were biasing the model.

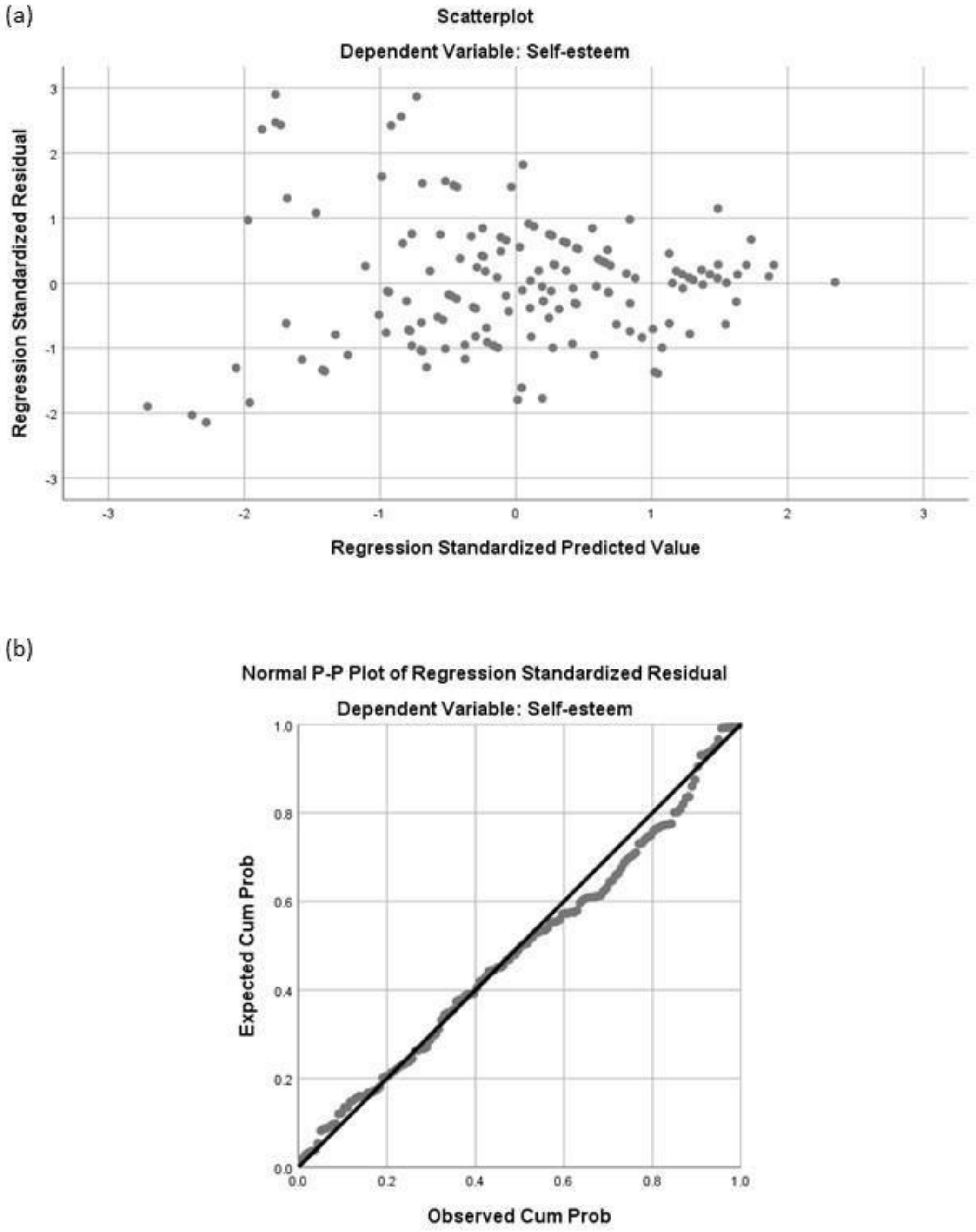

Figure 3. Assumption tests for multiple linear regression analysis of self-esteem in AV patients. (a) Scatterplot showing the assumption of homoscedasticity was not violated. **(b)** P-P plot showing the values of residuals were normally distributed.

Multiple linear regression analysis for QoL in AV patients

Assumptions

A collinearity diagnostic was carried out to confirm that there was no multicollinearity, and the tolerance and variance inflation factor (VIF) values did not indicate a violation of this assumption (Table 6). In order to evaluate the assumption that the values of the residuals are independent, the Durbin-Watson statistic was calculated, and this result revealed that the assumption was not violated (CADI: 1.827, DLQI: 1.906, WHOQoL: 1.498). Scatterplots indicated that the variance of the residuals was constant (homoscedasticity) (Fig. 4a-c), and P-P plots ensured that the values of residuals were normally distributed (Fig. 5a-c). Cook's Distance values were calculated to ensure that no influential cases were biasing the model. All values were below 1, suggesting that no cases were biasing the model.

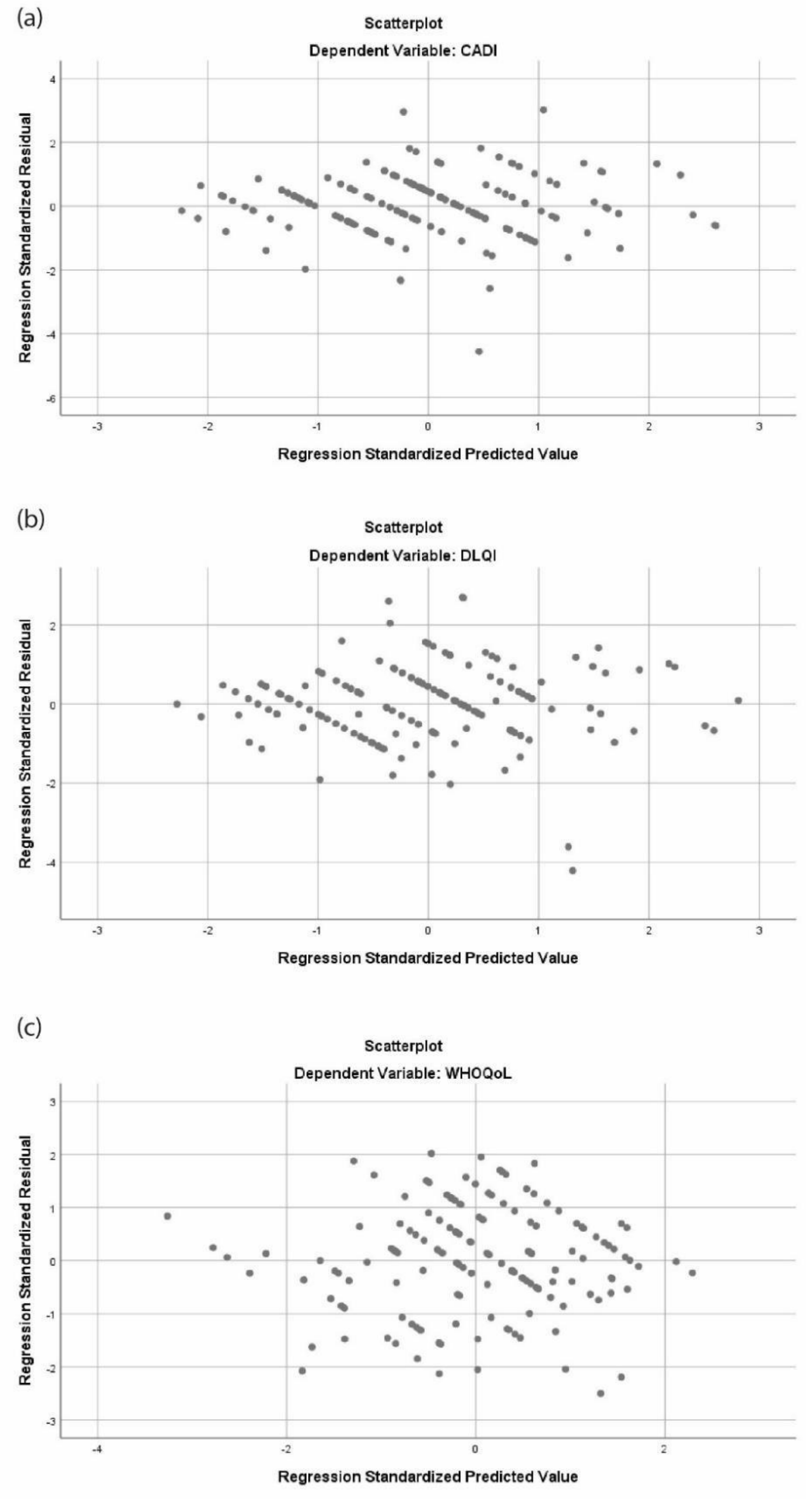

Figure 4. Assumption tests for multiple linear regression analysis of QoL in AV patients. Scatterplots showing the assumption of homoscedasticity were not violated for QoL scales: (a) CADI, (b) DLQI, and (c) WHOQoL.

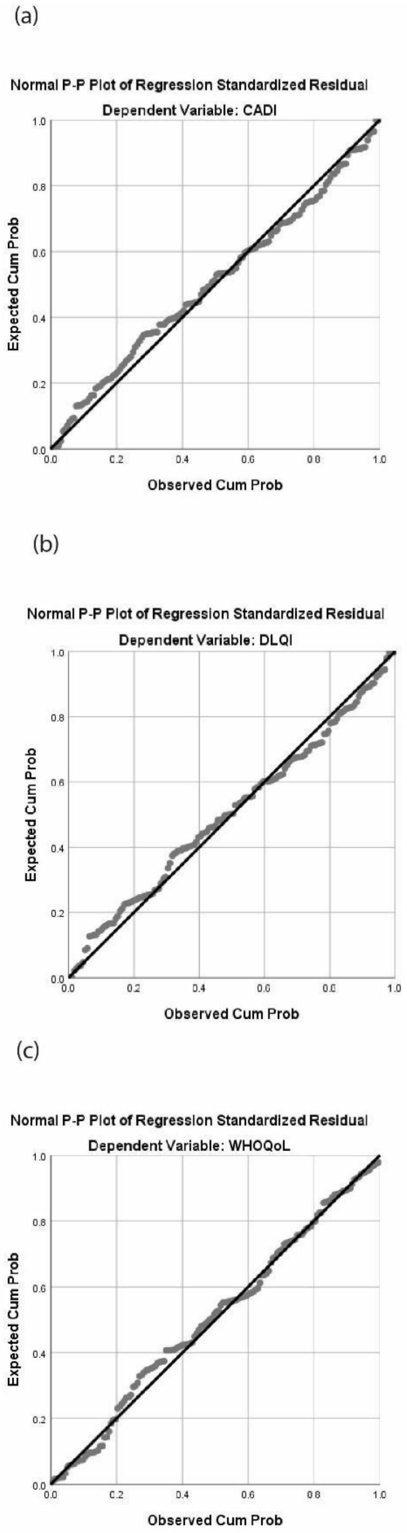

Figure 5. Assumption tests for multiple linear regression analysis of QoL in AV patients. P-P plots showing the values of residuals were normally distributed for all three QoL scales: (a) CADI, (b) DLQI, and (c) WHOQoL.
